# Complete Genome Sequencing and Comparative Genomic Analysis of *Helicobacter Apodemus* Isolated From the Wild Korean Striped Field Mouse *(Apodemus agrarius*) for Potential Pathogenicity

**DOI:** 10.3389/fphar.2018.00838

**Published:** 2018-07-31

**Authors:** Junhyung Kim, Woohyun Kim, Jae-Uk An, Jun Gyo Suh, Je Kyung Seong, Bo-Young Jeon, Seongbeom Cho

**Affiliations:** ^1^BK21 PLUS Program for Creative Veterinary Science Research, Research Institute for Veterinary Science and College of Veterinary Medicine, Seoul National University, Seoul, South Korea; ^2^Department of Medical Genetics, College of Medicine, Hallym University, Chuncheon, South Korea; ^3^Department of Biomedical Laboratory Science, College of Health Science, Yonsei University, Wonju, South Korea

**Keywords:** *Helicobacter apodemus*, korean striped field mouse, wild rodents, whole genome sequencing, comparative genomic analysis

## Introduction

The *Helicobacter* bacterial genus comprises of spiral-shaped gram-negative bacteria with flagella that colonize the gastro-intestinal (GI) tract of humans and various mammals (Solnick and Schauer, 2001). In particular, *Helicobacter pylori* was classified as a group 1 carcinogen by the International Agency for Research on Cancer (IARC) in 1994, and has been shown to occur with a high prevalence in humans, although this varies between geographical regions, ethnic groups, and various populations (Kusters et al., 2006; Goh et al., 2011). To date, more than 37 *Helicobacter* species have been identified in addition to *H. pylori* (Péré-Védrenne et al., 2017). Furthermore, non-*H. pylori* Helicobacters (NHPH) have been shown to infect both humans and animals, and NHPH infections are associated with intestinal carcinoma, and mucinous adenocarcinoma (Swennes et al., 2016). Despite the demonstrated association between NHPH and disease, most studies to date have investigated *H. pylori* in humans; thus, it is necessary to characterize NHPH and elucidate its role in the GI tract of wild rodents which are potential *Helicobacter* carriers (Taylor et al., 2007; Mladenova-Hristova et al., 2017).

*Helicobacter apodemus*, a spiral curved rod bacterium with a single flagella, was first identified in the GI tract of the Korean striped field mouse (*Apodemus agrarius*) in Korea, and shown to exhibit urease, oxidase, and catalase activity (Jeon et al., 2015). Since then, rodents colonized with *H. apodemus* have been found all over the world, including within the Xinjiang-Uygur Autonomous Region of China, Cambridge in the United States, and animal facilities in Sweden (Goto et al., 2004; Johansson et al., 2006; Miller et al., 2014). A previous study suggested that *H. apodemus* has the potential to cause rectal prolapse and colorectal cancer in rodents (Miller et al., 2014; Zhang et al., 2017), while another suggested that it may act as a rodent pathobiont, normally activating regulatory T-cells to maintain immune tolerance, but activating effector T-cells to contribute to inflammation and disease pathogenesis (Chai et al., 2017). Rodent *H. apodemus* colonization has been shown to be significantly decreased after treatment with azithromycin (compared to other antibiotics such as amoxicillin, or cefaclor), and similarly, after administration of *Lactobacillus casei* Zhang, and vitamin K2 (Khan et al., 2016; Zhang et al., 2017).

Nevertheless, continued research is essential to elucidate the molecular mechanisms by which *H. apodemus* alternately causes GI tract inflammation and GI tolerance in rodents, depending upon host health. The current study was therefore conducted to identify the genomic characteristics and specificity of *H. apodemus*, and to reveal its potential role in the rodent GI tract. Specifically, the genome of *H. apodemus* str. SCJK1 isolated from *Apodemus agrarius* was completely sequenced, and subjected to a comparative genomic analysis with 17 genome sequences of other *Helicobacter* species. It is hoped that the data in this study will serve as the basis for further studies of *H. apodemus-*related bacterium, and furthermore, enable future in-depth biomedical research regarding the immunological and pathological role of *H. apodemus* in the rodent GI tract.

## Materials and methods

### *H. apodemus* isolation and DNA extraction

In May 2015, fresh fecal samples from wild *A. agrarius* were collected, and transported to the laboratory at 4°C. The fecal samples were homogenized in PBS, spread onto modified Charcoal-Cefoperazone-Deoxycholate agar (mCCDA) with a selective supplement (Oxoid), and micro-aerobically incubated at 42°C for 6 days. After incubation, suspected colonies were transferred to blood agar, and micro-aerobically incubated at 42°C for 2 days. Genomic DNA was extracted from each colony confirmed to be *H. apodemus* (via Polymerase Chain Reaction (PCR) (Miller et al., 2014), and 16S rRNA sequencing analyses) using MG^TM^ Genomic DNA Purification kit (Macrogen, Korea). The quality of the extracted genomic DNA was evaluated using a 2100 Bioanalyzer (Agilent Technologies, Santa Clara, CA, USA).

### Whole-genome sequencing, genome assembly, and gene annotation

The whole-genome sequencing of *H. apodemus* str. SCJK1 was carried out using a PacBio RS α sequencer (Pacific Biosciences, Menlo Park, CA, USA). A 20 kb DNA library was prepared using a SMRTbell^TM^ template Prep Kit, and sequenced using a P6-C4 chemistry reagent kit (Pacific Biosciences, Menlo Park, CA, USA). The obtained sub-reads were assembled *de novo* using Hierarchical Genome Assembly Process v. 3.0 and SMRT Analysis v. 2.3 (default options) software (Pacific Biosciences, Menlo Park, CA, USA) (Chin et al., 2013). The reads were polished using Quiver v. 1.0 software (Pacific Biosciences, Menlo Park, CA, USA) to ensure a higher level of accuracy and lower error rate (Chin et al., 2013). Genes were annotated according to the National Center for Biotechnology Information (NCBI) Prokaryotic Genome Annotation Pipeline (PGAP, https://www.ncbi.nlm.nih.gov/genome/annotation_prok/), and “Clusters of Orthologous Group (COG)” categories were assigned using the NCBI COGs database (2014 version, https://www.ncbi.nlm.nih.gov/COG/). A summary of the generated sequencing data is included in Supplementary Table 1. Sequences for *Helicobacter*-related virulence genes presented in the Virulence Factor Database (VFDB, www.mgc.ac.cn/VFs/) were used to predict the *H. apodemus* str. SCJK1 virulence factor.

### Comparative genomic analysis of *H. apodemus* with other *helicobacter* species

A total of 17 genome sequences of other *Helicobacter* spp. were obtained from the NCBI database (https://www.ncbi.nlm.nih.gov/genome/), and used to conduct a comparative genomic analysis, including JRPC_s (GCA_ 000765745.1), *H. himalayensis* (GCA_ 001602095.1), *H. mustelae* (GCA_ 000091985.1), *H. cinaedi* (GCA_ 000349975.1), *H. bilis* (GCA_ 001999985.1), *H. cetorum* (GCA_ 000259275.1), *H. acinonychis* (GCA_000009305.1), *H. felis* (GCA_000200595.1), *H. hepaticus* (GCA_000007905.1), *H. pullorum* (GCA_001298055.1), *H. typhlonius* (GCA_001460635.1), *H. rodentium* (GCA_ 000687535.1), *H. Canadensis* (GCA_000162575.1), JRPB_s (GCA_000765695.1), *H. trogontum* (GCA_000765905.1), and two *H. pylori* (GCA_001653455.1 and GCA_000013245.1) (Supplementary Table 2). The orthologous Average Nucleotide Identity (OrthoANI) algorithm was used to measure the genetic relatedness between *H. apodemus* str. SCJK1 and the other *Helicobacter* spp., and Unweighted Pair Group Method with Arithmetic Mean (UPGMA) dendrogram was constructed based on the OrthoANI value (Lee et al., 2016). Pan-genome Orthologous Groups (POGs) were determined using the BIOiPLUG Comparative Genomics Database (https://www.bioiplug.com/), and a heat map and UPGMA dendrogram were constructed based on these data (i.e., the presence/absence of a POG).

### Quality assurance

To ensure a pure culture, a single *H. apodemus* colony was transferred three times. Furthermore, contamination was excluded by comparing three 16S rRNA gene fragments found in the *H. apodemus* str. SCJK1 genome using EzBioCloud DB software (https://www.bioiplug.com/).

### Ethics approval

Animal experiments were approved by the Institutional Animal Care and Use Committee (IACUC) at Yonsei University Wonju Campus (YWC-151203-1).

## Results and discussion

### Characterization of *H. apodemus* genome features

**Figure 1:**
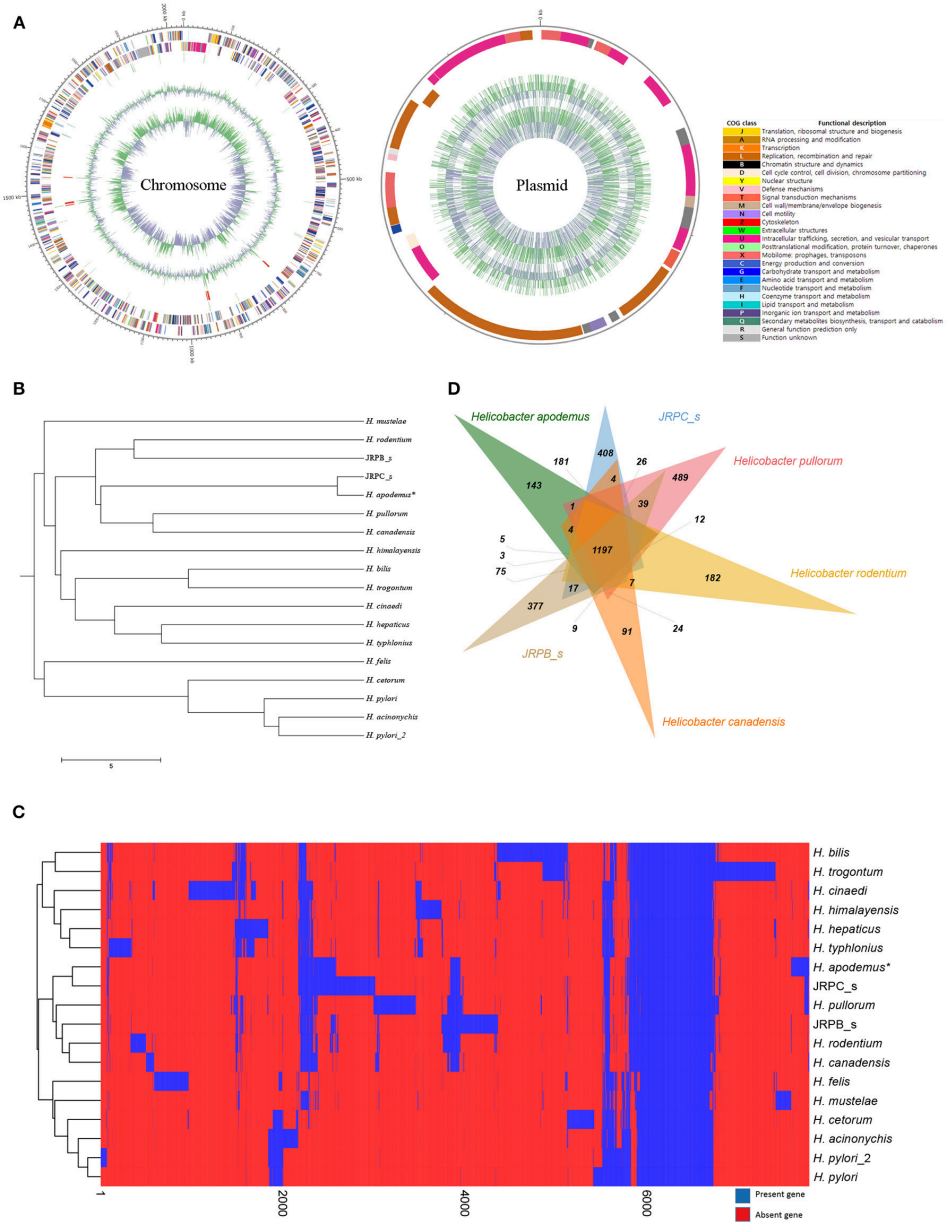
Complete genome sequence and comparative genomic analysis of *Helicobacter apodemus str*. SCJK1 with respect to 17 other *Helicobacter* spp. **(A)** Circular map of the *H. apodemus* str. SCJK1 genome, and Clusters of Orthologous Group (COG) categories within the genome, assigned using the NCBI COGs database. **(B)** Unweighted Pair Group Method with Arithmetic Mean (UPGMA) dendrogram of the analyzed *Helicobacter* spp. based on the orthologous Average Nucleotide Identity (Ortho ANI) value. **(C)** Heat map and UPGMA dendrogram of the analyzed *Helicobacter* spp. based on the presence or absence of Pan-genome Orthologous Groups (POG). **(D)** Venn diagram of six-clustered *Helicobacter* spp. based on POG analysis. ^*^H. apodemus str. SCJK1 genome used in the current study.

The complete genome of *H. apodemus* str. SCJK1 was shown to be composed of two circular contigs (one chromosome 2,034,706 bp in length, and one plasmid 33,248 bp in length) (Figure 1A), and to have an average GC content of 33.14% (chromosome, 33.2%; plasmid, 29.4%). Both size and GC content were found to be similar to those of the other analyzed *Helicobacter* spp., which were 1,886,022 ± 318,568 bp, and 37.3 ± 3.2%, respectively (mean ± standard deviation; Supplementary Table 2). Furthermore, the *H. apodemus* str. SCJK1 genome was identified to harbor a total of 1,850 coding, and 103 pseudo genes, as well as 38 transfer (tRNA), 9 ribosomal (rRNA; including three each of *5s, 16s*, and *23s* rRNA), and 3 non-coding (ncRNA) RNA sequences (Table 1). The various genes identified on the *H. apodemus* chromosome were assigned to COG categories, and thus predominantly classified as J (translation, ribosomal structure, and biogenesis, 8.27%), M (cell-wall/membrane/envelope biogenesis, 7.66%), R (general function prediction only, 7.00%), E (amino-acid transport and metabolism, 6.85%), C (energy production and conversion, 5.93%), and L (replication, recombination, and repair, 5.27%)-type genes. Moreover, the *H. apodemus* plasmid was annotated as predominantly carrying U (intracellular trafficking, secretion, and vesicular transport, 31.25%), L (18.75%), and X (mobilome: prophages, transposons, 12.5%)-type genes.

**Table 1:** Genomic features of *Helicobacter apodemus str*. SCJK1.

**Feature**	**Chromosome**	**Plasmid**	**Total**
Topology	Circular	Circular	–
Length (bp)	2,034,706	33,248	2,067,954
GC contents (%)	33.2	29.4	33.14
Number of genes (coding)	1,819	31	1,850
Number of tRNA	38	0	38
Number of rRNA	9	0	9
Number of ncRNA	3	0	3
CRISR arrays	1	0	1

### Virulence genes

In *Helicobacter* spp., a total of 91 virulence genes were identified, and classified into seven categories, and 17 subcategories. Specifically, these comprised of acid resistance (urease), adherence (*adherence-associated lipoprotein* (*alp*) A, *alp*B, blood group antigen binding adhesins, *H. pylori* adhesin (*hpa*) A, *hop*Z, *hor*B, *peb*1, and sialic acid binding adhesins), immune evasion (lipopolysaccharide Lewis antigens), immune-modulator (neutrophil-activating protein and outer inflammatory protein), motility (flagella), secretion system (cag pathogenicity island (*cag*-PAI)-type IV secretion system), and toxin (cytolethal distending toxin, vacuolating cytotoxin) genes (Supplementary Table 3). *H. apodemus* str. SCJK1 was found to harbor 47 of these 91 virulence genes, including all of the urease-related “acid resistance” genes, 30 of the 38 flagella-related “motility” genes, and three of the “toxin” genes. Of the 10 possible “adherence” genes, *H. apodemus* str. SCJK1 carried only *peb*1 (PEB1-related gene). In addition, the strain harbored only one (*fut*A) of the three “immune evasion” genes, and one (*nap*A) of the two “immune modulator” genes. The *H. apodemus* str. SCJK1 plasmid was shown to include the *Cag*X, V, E, and C (*vir*B 9, 8, 4, and 2, respectively) “secretion system” genes, known to be associated with the *cag*-PAI type IV secretion system.

Three *Helicobacter* strains, *H. acinonychis* str. Sheeba*, H. hepaticus* ATCC 51449, and *H. pylori* HPAG1, were used to infer virulence factors in the *H. apodemus* str. SCJK1 genome (Supplementary Table 3). Of the identified virulence genes, *peb*1 (“adherence” category), and *cdt*A and B (“toxin” category, and “cytolethal distending toxin” subcategory) were shown to be present in the *H. apodemus* and *H. hepaticus*, but not in the *H. pylori* genome (Tomb et al., 1997). Furthermore, *peb*1 has been previously reported to be expressed on the surface of all *Campylobacter jejuni* and *C. coli* bacteria, and to thereby mediate intestinal colonization, indicating that it is a prominent target for the immune system (Pei and Blaser, 1993). Consistent with these observations, *peb* 1 of *H. hepaticus* was expected to be involved in colonization of the intestine, and according to the results of this study, it may also be involved in intestinal colonization of *H. apodemus* (Suerbaum et al., 2003). In addition, the cytolethal distending toxin gene, consisting of *cdt*A, *cdt*B, and *cdt*C, has been shown to be expressed by GI pathogens including *Campylobacter* spp., *Escherichia coli*, and *Helicobacter* spp., and associated with irreversible G2/M cell-cycle arrest, which results in the gradual expansion of the nucleoli, and corresponding loss of the cytoplasm (Young et al., 2000; Taylor et al., 2003). Accordingly, *H. hepaticus*, which is known to carry the *cdt* gene, has been shown to be associated with chronic GI tract inflammation, and the onset of irritable bowel disease (IBD) in rodents (Whary and Fox, 2004; Young et al., 2004; Ge et al., 2007). The present study showed that *H. apodemus* also harbors the *cdt*A and *cdt*B genes; thus, further study should be conducted to investigate whether *H. apodemus* exerts similar impact on the rodent GI tract. In addition, both *H. pylori* and *H. apodemus* carry genes known to be associated with the *cag*-PAI type IV secretion system, responsible for horizontal gene transfer between bacterial cells (Rohde et al., 2003). *H. pylori* has been previously reported to mediate the pathogenesis of gastric adenocarcinoma and mucosa-associated lymphatic tissue (MALT) lymphoma, by injecting *cag*A (which is a bacterial gene that promotes cell proliferation and differentiation) into gastric epithelial cells using the *cag*-PAI type IV secretion system (Odenbreit et al., 2000; Cascales and Christie, 2003). However, the *H. apodemus* genome did not include *cag* A; thus, the role of the *cag*-PAI type IV secretion system in the *H. apodemus* genome should be studied further.

### Comparative genomic analysis

The genome of *H. apodemus* str. SCJK1 was compared with those of 17 other *Helicobacter* spp. In the constructed UPGMA dendrogram (based on orthoANI values), *H. apodemus* was shown to be not only closely related, but of the same species (orthoANI value 97.35) as JRPC_s, which was isolated from rats in the United States in 2003 (Lee et al., 2016). These results indicate that *H. apodemus* exists not only in *A. agrarius*, but also in other rodents, and also, demonstrate the low genetic difference between the isolates collected from the two different host types. Furthermore, *H. apodemus* was clustered with *H. pullorum* (isolated from fresh chicken meat in Portugal, 2012), *H. canadensis* (no isolation information), *H. rodentium* (isolated from a C57bl/6x129 mouse, 1995), and JRPB_s (isolated from a mouse in the United States, 2011), according to the calculated orthoANI values of 73.46, 73.52, 72.92, and 72.65, respectively (Figure 1B). The calculated relatedness between these species was consistent with the findings of previous studies, in which primers used in an *H. rodentium*-specific PCR assay cross-reacted with both *H. apodemus* and *H. rodentium* (Shen et al., 1997; Miller et al., 2014). However, the results of the present study were not consistent with the data presented in phylogenetic tree (based on 16S rRNA sequence comparisons) in which *H. apodemus* was clustered with *H. mesocricetorum, H. ganmani*, and *H. rodentium*, but not with *H. pullorum* or *H. canadensis* (Jeon et al., 2015).

In the heat map and UPGMA dendrogram (based on POG analysis), *H. apodemus* str. SCJK1 was clustered with JRPC_s, *H. pullorum, H. canadensis, H. rodentium*, and JRPB_s, consistent with orthoANI value-based results (Figure 1C). The consistency in results from orthoANI values-based and POG-based analysis indicated that *H. apodemus* was genetically similar to the above *Helicobacter* species (*H. pullorum, H. canadensis, H. rodentium*, and JRPB_s). In addition, the six strains shared 1,197 POGs, of which 143 were identified only in the strains analyzed in the present study (Figure 1D). *H. apodemus* strains (i.e., those isolated in the current study, and JRPC_s) shared 1,647 POGs, which were expected to have specific characteristics of *H. apodemus*.

## Future directions

Studies using whole-genome sequencing technology have made important advances in rodent GI microbial research; however, the present study is the first to analyze the complete genome sequence of *H. apodemus* from wild *A. agrarius*, which acts as a pathobiont in wild rodents. It is hoped that the results of this analysis, together with those of the conducted comparative genomic analysis, will serve as the basis for further biomedical studies investigating the immunological and inflammatory effects of *H. apodemus* on the rodent GI tract.

## Data access

The genome sequence of *Helicobacter apodemus* str. SCJK1 was deposited in the GeneBank under accession number CP021886-CP021887.

## Author contributions

SC conceived and designed the study. J-UA and WK analyzed the genome sequencing data. JGS, JKS, and B-YJ performed sampling, and prepared the manuscript. JK was a major contributor, both in experiments and writing the manuscript. All authors have read and approved the final manuscript.

### Conflict of interest statement

The authors declare that the research was conducted in the absence of any commercial or financial relationships that could be construed as a potential conflict of interest.
