# Supplementary material for: Complete Genome Sequencing and Comparative Genomic Analysis of Helicobacter Apodemus Isolated From the Wild Korean Striped Field Mouse (Apodemus agrarius) for Potential Pathogenicity
Source: Front Pharmacol. 2018 Jul 31;9:838. doi: 10.3389/fphar.2018.00838 (PMC6090156; doi:10.3389/fphar.2018.00838)
Supplement: Supplementary file 1 [file Table_1.docx]

Supplementary Material

**Complete genome sequencing and comparative genomic analysis of *Helicobacter apodemus* isolated from the wild Korean striped field mouse *(Apodemus agrarius*) for potential pathogenicity**

**Junhyung Kim^1^, Woohyun Kim^1^, Jae-Uk An^1^, Jun Gyo Suh^2^, Je Kyung Seong^1^, Bo-Young Jeon^3^, Seongbeom Cho^1*^**

^1^BK21 PLUS Program for Creative Veterinary Science Research, Research Institute for Veterinary Science and College of Veterinary Medicine, Seoul National University, Seoul, Republic of Korea

^2^Department of Medical Genetics, College of Medicine, Hallym University, Chuncheon, Korea

^3^Department of Biomedical Laboratory Science, College of Health Science, Yonsei University, Wonju 26493 Republic of Korea

*** Correspondence:** Dr. Seongbeom Cho

[chose@snu.ac.kr](mailto:chose@snu.ac.kr)

# Supplementary Tables

**Supplementary Table 1.** Summary of sequencing data of *Helicobacter apodemus* str. SCJK1

**Supplementary Table 2.** The 17 *Helicobacter* spp. that were used in the comparative genomic analysis

**Supplementary Table 3.** Comparison of virulence genes between *H. apodemus* str. SCJK1, *H. acinonychis* str. Sheeba*,* *H. hepaticus* ATCC 51449, and *H. pylori* HPAG1

**Supplementary Table 1.** Summary of sequencing data of *Helicobacter apodemus* str. SCJK1

| Properties | |
| --- | --- |
| Type of Sequencer | PacBio RS II |
| Library | PacBio SMRT bell^TM^ library |
| Gene assembly method | RS HGAP Assembly v. 3.0 |
| Gene annotation pipeline | NCBI Prokaryotic Genome Annotation Pipeline v. 4.2 |
| Assembly and annotation date | June-2017 |
| Genome coverage | Chromosome : 482 X  Plasmid : 181 X  Average genome coverage : 477.0 X |
| Number of reads | 132,129 |
| Number of contigs | 2 (1 chromosome and 1 plasmid) |
| Length of contigs | Chromosome : 2,034,706 |
|  | Plasmid : 33,248 |
|  | Total length : 2,067,954 |
| Accession number | CP021886-CP021887 |

**Supplementary Table 2.** The 17 *Helicobacter* spp. that were used in the comparative genomic analysis

|  | Assembly ID | Taxon Name | Length  (Mb) | GC contents (%) | No. of CDS |
| --- | --- | --- | --- | --- | --- |
| 1 | GCA_000765745.1 | JRPC_s | 2.11494 | 33% | 2,596 |
| 2 | GCA_001602095.1 | *Helicobacter himalayensis* | 1.82994 | 39.9% | 1,711 |
| 3 | GCA_000091985.1 | *Helicobacter mustelae* | 1.5781 | 42.5% | 1,421 |
| 4 | GCA_000349975.1 | *Helicobacter cinaedi* | 2.21447 | 38.5% | 2,328 |
| 5 | GCA_001999985.1 | *Helicobacter bilis* | 2.50811 | 34.9% | 2,324 |
| 6 | GCA_000259275.1 | *Helicobacter cetorum* | 1.90395 | 35.0% | 1,730 |
| 7 | GCA_000009305.1 | *Helicobacter acinonychis* | 1.55759 | 38.2% | 1,547 |
| 8 | GCA_000200595.1 | *Helicobacter felis* | 1.67268 | 44.5% | 1,671 |
| 9 | GCA_000007905.1 | *Helicobacter hepaticus* | 1.79915 | 35.9% | 1,797 |
| 10 | GCA_001298055.1 | *Helicobacter pullorum* | 1.81417 | 34.3% | 2,193 |
| 11 | GCA_001460635.1 | *Helicobacter typhlonius -* | 1.83057 | 38.9% | 1,912 |
| 12 | GCA_000687535.1 | *Helicobacter rodentium* | 1.81065 | 37% | 1,817 |
| 13 | GCA_000162575.1 | *Helicobacter Canadensis* | 1.62867 | 33.8% | 1,574 |
| 14 | GCA_000765695.1 | JRPB_s | 1.89979 | 36.9% | 2,152 |
| 15 | GCA_000765905.1 | *Helicobacter trogontum* | 2.65793 | 33.15% | 2,654 |
| 16 | GCA_001653455.1 | *Helicobacter pylori* | 1.63593 | 38.9% | 1,482 |
| 17 | GCA_000013245.1 | *Helicobacter pylori* | 1.60574 | 39.1% | 1,507 |

**Supplementary Table 3.** Comparison of virulence genes between *H. apodemus* str. SCJK1, *H. acinonychis* str. Sheeba*,* *H. hepaticus* ATCC 51449, and *H. pylori* HPAG1

| Virulence factors | | genes | *H. apodemus str.* SCJK1 | *H. acinonychis* str. Sheeba | *H. hepaticus* ATCC 51449 | *H. pylori* HPAG1 |
| --- | --- | --- | --- | --- | --- | --- |
| Acid resistance | Urease | *ure*A | + | + | + | + |
|  |  | *ure*B | + | + | + | + |
|  |  | *ure*I | + | + | + | + |
|  |  | *ure*E | + | + | + | + |
|  |  | *ure*F | + | + | + | + |
|  |  | *ure*G | + | + | + | + |
|  |  | *ure*H | + | + | + | + |
| Adherence | adherence-associated lipoprotein AlpA | *alp*A/*hop*C | - | + | - | + |
|  | AlpB | *alp*B/*hop*B | - | + | - | + |
|  | Blood group antigen binding adhesins | *bab*A/*hop*S | - | - | - | + |
|  |  | *bab*B/*hop*T | - | - | - | + |
|  | *H. pylori* adhesin A | *hpa*A | - | + | - | + |
|  | HopZ | *hop*Z | - | - | - | + |
|  | HorB | *hor*B | - | + | - | + |
|  | PEB1 | *peb*1 | + | - | + | - |
|  | Sialic acid binding adhesins | *sab*A/*hop*P | - | + | - | + |
|  |  | *sab*B/*hop*O | - | - | - | + |
| Immune evasion | Lipopolysaccharide Lewis antigens | *fut*A | + | + | + | + |
|  |  | *fut*B | - | + | - | + |
|  |  | *fut*C | - | + | + | + |
| Immune modulator | Neutrophil-activating protein (HP-NAP) | *nap*A | + | + | + | + |
|  | Outer inflammatory protein | *oip*A/*hop*H | - | + | - | + |
| Motility | Flagella | *fla*A | + | + | + | + |
|  |  | *fla*B | + | + | + | + |
|  |  | *fla*G | - | + | + | + |
|  |  | *fli*R | - | + | + | + |
|  |  | *flg*I | + | + | + | + |
|  |  | *flg*L | + | + | + | + |
|  |  | *flg*H | + | + | + | + |
|  |  | *fla*G | - | + | - | + |
|  |  | *fli*F | + | + | + | + |
|  |  | *fli*G | + | + | + | + |
|  |  | *fli*H | + | + | + | + |
|  |  | *flg*G_1 | + | + | + | + |
|  |  | *flh*A | + | + | + | + |
|  |  | *flh*F | + | + | + | + |
|  |  | *fli*A | + | + | + | + |
|  |  | *fli*M | + | + | + | + |
|  |  | *fli*Y | - | + | + | + |
|  |  | *fli*N | + | + | + | + |
|  |  | *fli*P | + | + | + | + |
|  |  | *fli*D | + | + | + | + |
|  |  | *fli*S | + | + | + | + |
|  |  | *flh*B_1 | + | + | + | + |
|  |  | *fli*L | - | + | + | + |
|  |  | *mo*tA | + | + | + | + |
|  |  | *mo*tB | + | + | + | + |
|  |  | *flg*E_1 | + | + | + | + |
|  |  | *flg*D | + | + | + | + |
|  |  | *flg*E_2 | + | + | + | + |
|  |  | *flg*K | + | + | + | + |
|  |  | *pfl*A | - | + | + | + |
|  |  | *fli*Q | - | + | + | + |
|  |  | *fli*I | + | + | + | + |
|  |  | *flg*A | - | + | - | + |
|  |  | *fli*E | + | + | + | + |
|  |  | *flg*C | + | + | + | + |
|  |  | *flg*B | + | + | + | + |
|  |  | *flh*B_2 | + | + | + | + |
|  |  | *flg*G_2 | + | + | + | + |
| Secretion system | Cag PAI type IV secretion system | *cag*1 | - | - | - | + |
|  |  | *cag*2 | - | - | - | + |
|  |  | *cag*3 | - | - | - | + |
|  |  | *cag*4 | - | - | - | + |
|  |  | *cag*5 | - | - | - | + |
|  |  | *vir*B11 | - | - | - | + |
|  |  | *cag*Z | - | - | - | + |
|  |  | *cag*Y | - | - | - | + |
|  |  | *cag*X | + | - | - | + |
|  |  | *cag*W | + | - | - | + |
|  |  | *cag*V | + | - | - | + |
|  |  | *cag*U | - | - | - | + |
|  |  | *cag*T | - | - | - | + |
|  |  | *cag*S | - | - | - | + |
|  |  | *cag*Q | - | - | - | + |
|  |  | *cag*P | - | - | - | - |
|  |  | *cag*M | - | - | - | + |
|  |  | *cag*N | - | - | - | + |
|  |  | *cag*L | - | - | - | + |
|  |  | *cag*I | - | - | - | + |
|  |  | *cag*H | - | - | - | + |
|  |  | *cag*G | - | - | - | + |
|  |  | *cag*F | - | - | - | + |
|  |  | *cag*E | + | - | - | + |
|  |  | *cag*D | - | - | - | + |
|  |  | *cag*C | + | - | - | + |
|  | T4SS effectors cytotoxin-associated gene A | *cag*A | - | - | - | + |
| Toxin | Cytolethal distending toxin | *cdt*A | + | - | + | - |
|  |  | *cdt*B | + | - | + | - |
|  |  | *cdt*C | - | - | + | - |
|  | Vacuolating cytotoxin | *vac*A | + | + | - | + |

+: This means that strain has genes.

-: This means that strain does not have genes.
